# Sexual Consent Perceptions of a Fictional Vignette: A Latent Growth Curve Model

**DOI:** 10.1007/s10508-021-02048-y

**Published:** 2021-11-10

**Authors:** Malachi Willis, Kristen N. Jozkowski

**Affiliations:** 1 MRC/CSO Social and Public Health Sciences Unit, Institute of Health and Wellbeing, University of Glasgow, Glasgow, G12 8QQ UK; 2 Department of Applied Health Science, School of Public Health, and the Kinsey Institute for Research in Sex, Gender, and Reproduction, Indiana University, Bloomington, IN USA

**Keywords:** Sexual consent, Perceptions, Experimental design, Latent growth curve, Structural equation modeling

## Abstract

Sexual consent can be conceptualized as a process of accumulating cues that build toward and continue throughout a consensual sexual encounter. How people perceive the cues of others during this process is an important aspect of consent. However, previous research has not investigated the trajectories of people’s consent perceptions throughout such a process. Using a novel staggered vignette protocol, we examined participants’ (*N* = 1218; 64.4% female) perceptions of fictional targets’ sexual consent at 11 time points. We tested latent growth curve models using multilevel structural equation modeling to examine trajectories in consent perceptions over the course of the vignette. We hypothesized that mean differences and rates of change would be associated with several constructs relevant to sexual consent. We found that initial consent perceptions and trends over the course of the vignette varied by whether the participant was a university student, by an alcohol manipulation in the vignette, by the fictional target’s sex, and by type of sexual behavior. Researchers should examine whether our findings on consent perceptions of a fictional vignette extend to people’s actual sexual encounters, including potential associations between the three primary aspects of sexual consent: perceptions, feelings, and communication.

## Introduction

Investigating how people perceive sexual consent can help understand consensual sexual encounters. A recent review of the sexual consent literature identified and described three prevailing conceptualizations of sexual consent: consent as an internal feeling, consent as external communication, and consent as a perception of someone else’s willingness (Muehlenhard et al., 2016). First, sexual consent can be conceptualized as an unobservable internal state of willingness (Jozkowski, Sanders, et al., 2014). Because internal feelings of consent are intangible, laws, policies, and many researchers are not keen to prioritize this definition of consent; instead, they emphasize the words and behaviors—explicit or implicit—that may be used to try to communicate or infer willingness (Hickman & Muehlenhard, 1999). As such, sexual consent may also be conceptualized as an act of agreeing to sexual activity, which might include behaviors ranging from passionate kissing to intercourse. Explicit communication involves a person clearly and unambiguously communicating to another person that they agree to engage in a sexual behavior, while implicit communication suggests agreement via indirect signals that can either be active or passive (Muehlenhard et al., 2016). Finally, people need to infer from the explicit or implicit communicative cues of others—or from cues based on context—to determine whether another person is willing. In the present study, we focused on this last aspect: perceptions of others’ sexual consent.

Measuring consent perceptions as a sexual encounter unfolds is important because series of sexual consent cues tend to precede consensual sexual behaviors. Muehlenhard et al. (2016) suggested that the accumulation of these cues increases the probability that a person is willing to engage in a sexual interaction. In other words, sexual consent can be conceptualized as an ongoing and iterative process that builds toward and continues throughout a consensual sexual encounter (Beres, 2010, 2014; Humphreys, 2004). Sequential behaviors that are part of this process may be observed and interpreted rapidly or over longer periods of time in a continuous fashion (Muehlenhard et al., 2016). Therefore, consent does not seem to be a one-time event that immediately precedes a sexual behavior; indeed, the process of consent can begin as early as interactions in social settings (Jozkowski et al., 2018). However, the extant empirical literature has scarcely examined patterns of consent perceptions or trajectories inherent to this ongoing process (Beres, 2007; Muehlenhard et al., 2016).

Consent as a continuous and cumulative process primarily relies on nonverbal cues or implicit verbal cues (e.g., asking someone to leave a social setting to go to a private setting). Explicitly and verbally communicating consent each time someone slightly moves would be “onerous and unrealistic” (Muehlenhard et al., 2016, p. 476). People are diverse in how they communicate their consent nonverbally. Implicit nonverbal consent cues might include touching somebody’s hand and arm or smiling; explicit nonverbal consent cues could be lifting hips for somebody to take off underwear or presenting one’s partner with a condom (e.g., Beres, 2010; Jozkowski, Peterson, et al., 2014; Jozkowski, Sanders, et al., 2014). Several different communicative cues likely precede consensual sexual behavior—each one may increase the probability that people perceive somebody to be willing to engage in sexual activity (Beres, 2010, 2014; Jozkowski et al., 2018; Muehlenhard et al., 2016).

Contextual cues also contribute to whether people perceive sexual behavior to be consensual. Rather than being communicated by either person, these cues reflect settings or situations wherein people are more likely to assume that others are willing to engage in sexual activity. Situational sexual consent cues identified in previous research include being alone with somebody in a private setting (Beres, 2010) and the presence of alcohol (Jozkowski et al., 2018). Interpersonal contexts can influence people’s consent perceptions as well (Humphreys, 2007; Marcantonio et al., 2018). Simply being in a committed relationship with somebody can be perceived as a consent cue (O’Byrne et al., 2008; Willis & Jozkowski, 2019). These contexts in which people interact can influence the probability that somebody is perceived to be willing to engage in sexual activity.

Validly collecting data on the development of sexual consent perceptions during people’s own sexual experiences would understandably be difficult, and retrospective self-reports of sexual behavior are subject to memory biases (Horvath et al., 2007; Willis & Jozkowski, 2018). However, a less invasive methodology has been used in previous studies to investigate consent perceptions. Specifically, researchers have presented people with vignettes (or stories) that detail a sexual encounter and then asked them questions regarding the fictional targets’ willingness to engage in sexual activity (e.g., Humphreys, 2007; Lofgreen et al., 2017; Margolin, 1990; Osman, 2003). This type of methodology allows researchers to control the cues—communicative or contextual—that participants can use to perceive sexual consent. For example, Humphreys’ (2007) vignette study depicted a heterosexual couple eating dinner, watching a movie, and engaging in sexual behavior; further, relationship history was manipulated: the fictional targets were on a first date with no sexual history, dating for three months with limited sexual history, or married for two years with more sexual history. After reading the entire vignette, participants were asked questions that measured perceptions of sexual consent, such as whether they agreed that “[the target’s] nonverbal behaviors clearly indicate that [she/he] has consented to sexual activity” (Humphreys, 2007, p. 310). To our knowledge, previous vignette studies have not assessed trajectories of consent perceptions because participants were presented the entire story before reporting their consent perceptions. We sought to overcome this limitation by examining consent perceptions at multiple time points to account for the continuous process that builds toward and continues throughout a consensual sexual encounter.

### Present Study

The goal of the present study was to examine how perceptions of targets’ sexual consent change as participants learn more about a fictional consensual sexual encounter. We developed a staggered vignette protocol that presented a limited amount of information to participants and asked them to evaluate the targets’ willingness to engage in sexual behavior before presenting them with additional information. Based on Muehlenhard et al.’s (2016) proposed probabilistic model of sexual consent, we assessed the continuous process of developing consent perceptions over the course of a fictional sexual encounter. Further, we examined whether several constructs that have been implicated in previous research on sexual consent influenced this process. We made hypotheses based on six of these constructs.

First, we compared female and male participants because sex consistently discriminates sexual consent perceptions. Studies have shown that males are more likely than females to perceive targets in vignettes as willing to engage in sexual activity (e.g., Humphreys, 2007; Margolin, 1990). Compared with females, males also interpret a wider variety of cues as indicating sexual consent or sexual interest and are more likely to conceptualize consent as a discrete event—rather than a larger process (Beres, 2010; Jozkowski et al., 2018; Perilloux et al., 2012). Because males more readily assume a person’s consent, we hypothesized that male participants would initially perceive the targets as more willing to engage in sexual activity than female participants. Further, we expected the rate of change in consent perceptions to be greater for male participants compared with female participants because males are more likely to interpret single cues as consent.

Second, we compared university students and non-students. Much of the attention regarding sexual consent has been focused on college campuses—likely in response to the publicity that campus sexual assault has received. Bennett (2016) estimated 1500 college campuses in the USA had adopted policies or standards that encourage explicit consent communication. Corresponding affirmative consent education initiatives have instructed college student populations to not interpret implicit and nonverbal cues as consent (Beres, 2014; Curtis & Burnett, 2017). However, comparisons between students and non-students regarding consent perceptions have been scarce; non-students are under-represented in much of the academic literature on sexual consent. In a systematic review of the consent literature, Willis et al. (2019b) reported that 85% of samples used to investigate sexual consent consisted entirely of college students and several others primarily comprised college students. Because students may be more reluctant to perceive someone’s consent in the absence of explicit communication, we hypothesized that non-students would initially perceive the targets as more willing to engage in sexual activity than university students. Further, we expected the rate of change in consent perceptions to be greater for non-students compared with university students because non-students may be more likely to interpret implicit and nonverbal consent cues as consent. We also predicted that these effects would be significant even when controlling for participants’ ages.

Third, we manipulated whether an offer of an alcoholic drink was accepted or rejected by one of the targets in our vignettes. According to previous research, people sometimes perceive accepting an alcohol as a sexual consent cue (Jozkowski, et al., 2018; Muehlenhard et al., 2016). While there has been debate regarding the ability to consent while under the influence of alcohol (Ward et al., 2012), people overwhelmingly believe that their intoxicated friend could consent to sexual activity disregarding how many drinks that friend seemed to have consumed (Drouin et al., 2019). As such, alcohol consumption may promote consent perceptions more than it impedes them. For these reasons, we predicted that participants would perceive the targets as more willing to engage in sexual activity when one target had accepted a drink from the other compared with those in a non-alcohol condition. Because this manipulation was only present in the first vignette segment, we did not have a directional hypothesis regarding group differences in rate of change in consent perceptions over time.

Fourth, we manipulated the interpersonal context of the targets in our vignettes. Indeed, the nature of a relationship can influence whether people perceive it permissible for one partner to assume another is interested in and consenting to sexual activity (Beres, 2014; Righi et al., 2019). Using vignettes, Humphreys (2007) found that scenarios indicating a relatively more romantic relationship between targets were perceived as clearer in sexual intent, more acceptable, less in need of additional precautions, and overall more consensual (Humphreys, 2007). In the present study, we sought to extend the already established effects of being in a committed relationship on sexual consent (Humphreys, 2007; Marcantonio et al., 2018; Willis, et al., 2019b) by examining the potential variation of interpersonal history within the context of a first-time casual sexual encounter—having just met versus being acquaintances. Because people are more likely to assume consent between acquaintances than strangers in nonconsensual vignettes (Bridges, 1991; Check & Malamuth, 1983), we predicted that participants in an acquaintance condition would initially perceive the targets as more willing to engage in sexual activity than those in a just met condition. This manipulation was also only present in the first vignette segment, so we did not have a directional hypothesis regarding group differences in rate of change in consent perceptions over time.

Fifth, we measured participants’ consent perceptions for both female and male targets. Previous research has indicated that females are perceived to be hesitant and males are assumed to always want sex (Jozkowski & Peterson, 2013; Jozkowski et al., 2017). Based on these stereotypically gendered roles, both females and males are prone to assuming a male’s sexual consent (Hirsch et al., 2019; Righi et al., 2019). Because people tend to perceive males as more willing than females to engage in sexual activity or think that males are always willing, we hypothesized that participants would initially perceive the male target as more willing to engage in sexual activity than the female target. Further, we expected the rate of change in consent perceptions to be greater when assessing the male target compared with the female target because participants may be more likely to interpret individual cues as consent for male targets compared with female targets.

Finally, we measured participants’ consent perceptions for several types of sexual behavior. Previous research has indicated that a highly relevant context to consider for within-person variability of sexual consent is type of sexual behavior one is engaging in (Marcantonio et al., 2018; Willis, et al., 2021a). For example, the proportion of students who believe that explicit consent is necessary increases as the perceived level of intimacy of the sexual behavior increases (Humphreys, 2007; Jozkowski, Sanders, et al., 2014). Because there is an established sexual script that consent does not need to be explicitly communicated for sexual behaviors that are lower-order, we hypothesized that participants would initially perceive the targets as more willing to engage to engage in less intimate behaviors compared with more intimate behaviors. Based on previous research (e.g., Sanders et al., 2010; Willis et al., 2020), making out—or passionate kissing—was considered to be the least intimate, followed by genital touching, oral sex, vaginal sex, and anal sex. Further, we expected the rate of change in consent perceptions to be greater when assessing relatively less intimate behaviors because fewer implicit or nonverbal cues might be required for participants to perceive the targets as willing to engage in less intimate sexual behaviors.

## Method

### Participants

In sum, 1615 people started this study; 361 participants were removed for missing data, 26 for potentially exhibiting inattention (detailed below), nine for identifying as a non-binary sex, and one for not meeting the eligibility criterion of being at least 18 years old. Thus, our analytic sample comprised 1218 people. Participants were 27.2 years old on average (SD = 12.3; range = 18–85), and 31.8% (*n* = 387) were not university students. Most participants identified as female (*n* = 784; 64.4%). Females tended to be exclusively or predominantly sexually attracted to males (*n* = 690; 88.1%); males tended to be exclusively or predominantly sexually attracted to females (*n* = 343; 79.2%). About half of the participants were in a committed relationship at the time of the study (*n* = 614; 50.5%). Regarding racial/ethnic identity, 83.0% were White (*n* = 1011), 6.8% were Black (*n* = 83), 5.3% were Hispanic (*n* = 65), 5.0% were Asian (*n* = 61), and 5.3% (*n* = 64) identified as another race/ethnicity.

### Procedure

Anybody at least 18 years old was eligible to participate. From spring 2017 to fall 2019, participants were recruited via social media, word-of-mouth, or course instructors to “complete an online survey about fictional sexual experiences.” Those recruited via social media or word-of-mouth were not given incentives to participate. Those participants recruited by instructors were enrolled in at least one of several general education courses at a large public university in the southern USA and were offered extra credit for their participation. As an alternative to participating in the study for extra credit, students were also offered the opportunity to complete a separate extra credit assignment not related to research participation. All interested people accessed the study online via Qualtrics Survey Software and provided their informed consent before participating in the study. After first filling out sociodemographic items, participants were randomly assigned a vignette that described a sexual encounter. The vignette was presented in 11 discrete segments. After each segment, participants indicated how willing to engage in various sexual behaviors they perceived each target to be. All study procedures were approved by the institutional review board.

#### Vignette Development

To examine participants’ perceptions of fictional targets’ sexual consent, we developed a vignette of a consensual sexual encounter between a female target and a male target that presented information in a staggered manner (see “Appendix” for complete vignettes). Informed by previous research and consultations with three outside content experts on sexual consent, we included a variety of cues that people perceive as indicating consent; these cues were both communicative and contextual. Communicative cues demonstrated in the vignette were either implicit or explicit (Hickman & Muehlenhard, 1999; Jozkowski, Peterson, et al., 2014; Jozkowski, Sanders, et al., 2014). We exclusively included nonverbal cues, which are most commonly used and are the type of cue primarily relied on when conceptualizing consent as a continuous process (Muehlenhard et al., 2016). Contextual consent cues in the vignette included being in a private setting (Jozkowski et al., 2018), an alcohol manipulation (Jozkowski et al., 2018), and an interpersonal context manipulation (Marcantonio et al., 2018). See Table 1 for the communicative or contextual consent cues included in each segment of the vignette.

**Table 1:** Descriptive statistics for consent perceptions over the vignette progression (*N* = 1218)

Segment	Consent Cue	*M*	SD
1st	Getting along	3.71	1.08
2nd	Flirtatious touching	4.30	1.06
3rd	Transition to private setting	4.82	1.06
4th	Legs touching	4.92	1.02
5th	Holding hands	5.14	0.96
6th	Mutual making out	5.53	0.85
7th	Removing shirts/Transition to bedroom	5.88	0.78
8th	Removing pants	5.98	0.70
9th	Butt lift for underwear removal	6.10	0.66
10th	Oral-genital stimulation	6.28	0.54
11th	Condom application/Sex begins	6.39	0.51

Consent perceptions were measured on a seven-point Likert-type scale from *Definitely not* to *Definitely*

In the first vignette segment, alcohol and interpersonal context were manipulated. Regarding alcohol, one target offered the other a drink in every condition; in randomly assigned conditions, the other target either accepted or rejected this drink. Regarding interpersonal context, randomly assigned conditions indicated that the targets either had either just met for the first time or had been friends for a few weeks. We systematically controlled for the targets’ sex associated with particular consent cues by alternating their names throughout the vignette and presenting each condition randomly; in all conditions, both targets were similarly initiating and responding to sexual consent cues. Therefore, we had four randomly assigned experimental conditions: alcohol/friends (*n* = 309); alcohol/just met (*n* = 292); no alcohol/friends (*n* = 307); no alcohol/just met (*n* = 310).

We piloted this vignette with undergraduate and graduate research assistants to improve its wording and believability. Regarding word choices throughout the vignette, research assistants helped select the terms for each of the sexual behaviors. For example, “making out” was interpreted as passionate kissing—consistent with definitions used in previous research (e.g., Marchi & Guendelman, 1994). In the present study, 84.4% of participants indicated that they thought the vignette was *Extremely believable* or *Moderately believable*. Generic US American names were used for the targets (i.e., Kim and Mike); both are short and contain similar letters. Because these names were paired with traditionally gendered pronouns, we removed participants (*n* = 26) who perceived Kim to be male or Mike to be female from the analytic sample as a potential attention check. Similar to our sample’s own sociodemographic characteristics, people tended to perceive these targets to be about 25 years old and White.

### Measures

#### Sociodemographic Variables

Participants reported their identity regarding a variety of sociodemographic variables: sex, age, student status, sexual attraction, relationship status, and race/ethnicity. Sex was assessed dichotomously (0 = male; 1 = female). Age was measured continuously in years. Student status was assessed dichotomously (0 = not a university student; 1 = currently a university student). Sexual attraction was measured on a seven-point scale (exclusively females to exclusively males); participants could also select (“I am not sexually attracted to females nor males”). Relationship status was measured nominally (i.e., single and not dating, single but casually dating, in a relationship, engaged or married). Race/ethnicity was measured nominally (i.e., Asian/Asian American, Black/African American, Hispanic/Latin American, Middle Eastern/Middle Eastern American, Native Hawaiian/Pacific Islander, White/European American, other race/ethnicity); participants selected all that applied.

#### Sexual Consent Perceptions

To measure participants’ perceptions of the targets’ sexual consent, we asked participants, “From the information provided, do you think [Kim/Mike] would be willing to engage in any of the following behaviors with [Mike/Kim]?” They responded on a seven-point scale: *Definitely not*, *No*, *Probably not*, *Not sure*, *Probably*, *Yes*, *Definitely*. Higher scores indicate perceiving that the targets were more likely to be willing to engage in a sexual behavior.

After each new segment of the vignette, participants reported how likely they thought the targets were willing to engage in sexual activity. They did this independently by sex of the target (i.e., female versus male) and by type of sexual behavior (i.e., making out, genital touching, oral sex, vaginal sex, and anal sex). Therefore, participants provided 10 consent perceptions after each of the 11 vignette segments was presented (i.e., 110 total ratings).

### Analysis

To examine how participants’ perceptions of sexual consent changed as they read about a fictional consensual sexual encounter, we tested latent growth curve models using multilevel structural equation modeling. We used the *lavaan* package of *R* to run these models (Rosseel, 2012). The primary advantage of latent growth curves over traditional statistical techniques based on ordinary least squares (e.g., analysis of variance [ANOVA]) is that they are able to model patterns of change when data are collected over multiple time points (Duncan et al., 2013). Further, observations in the present study violated the assumption of independence because multiple measures were assessed at multiple points for each participant. We accounted for this nested design by testing the effects of within-person variables using a clustering option available in the *lavaan* package. This analytic approach allowed us to control for within-person variability, which previous research has indicated is relevant for sexual consent (Willis et al., 2021a).

In each latent growth curve model, data from all 11 vignette segments were used to estimate intercepts and slopes. The intercepts represent scores at one point in time and can be set to a time point of interest; we chose the beginning of the present study (i.e., after the first vignette segment). The slopes represent the patterns or trajectories of change over time in the dependent variable (i.e., consent perceptions). We constructed both linear and quadratic effects to account for the nonlinear nature of our data.

First, we tested multiple unconditional models to determine the nature of the growth curve. Specifically, we tested a latent means model, a linear effect model, and a quadratic effect model. Second, we tested a conditional model that included the hypothesized independent variables. Between-person independent variables included the participant’s sex, the participant’s status as a university student, the alcohol manipulation, and the interpersonal context manipulation. Because participants who were not university students at the time of the study were significantly older than those who were, *t*(413) = 25.77, *p* < 0.001, we controlled for the effect of age as a continuous covariate in the model. Within-person independent variables included the target’s sex and the sexual behavior for which they were perceiving consent. Finally, in favor of parsimony, we then tested a reduced version of the hypothesized conditional model that retained only the significant predictors of the full model.

For the predicted paths, we reported standardized coefficients (*β*), unstandardized coefficients (B), and standard errors (SE). We also reported the statistical significance of the standardized coefficients (α = 0.05). A recommended minimum size for standardized coefficients that represents a practically significant effect for social science data is 0.2 (Ferguson, 2009).

Regarding data-model fit, we reported the χ^2^ value; non-significant χ^2^ values indicate ideal data-model fit. For the model to be considered as fitting the data well when the χ^2^ value is not significant, Hu and Bentler (1999) recommended that the comparative fit index (CFI) be greater than 0.95, the root-mean-square error of approximation (RMSEA) less than 0.06, and the standardized root-mean-square residual (SRMR) less than 0.08. An advantage of the SRMR for multilevel models is that it distinguishes data-model fit for the between- and within-person effects. We reported each of these fit statistics.

## Results

Table 1 provides means and standard deviations for sexual consent perceptions over the 11 segments of vignette progression. The latent growth curve models examined changes in consent perceptions and predictors of that change. The unstandardized coefficients, standard errors, and standardized confidents with their significance indicated for the unconditional and conditional models (described in detail below) are presented in Table 2. In the final portion of Table 2, model fit indices are reported to assess how well the latent growth curve models fit the data.

**Table 2:** Multilevel latent growth curve models predicting consent perceptions (*N* = 1218)

	Unconditional model		Conditional model: full		Conditional model: reduced	
	*β*	*B* (SE)	*β*	*B* (SE)	*β*	*B* (SE)
Overall intercept	2.58***	3.87 (0.01)	5.10***	5.68 (0.19)	4.85***	5.39 (0.06)
Overall linear effect	1.01***	0.42 (0.00)	1.99***	0.55 (0.05)	2.13***	0.59 (0.02)
Overall quadratic effect	− 0.51***	− 0.02 (.00)	− 1.53***	− 0.03 (.00)	− 1.59***	− 0.03 (0.00)
Intercept regressed onto						
Alcohol (between)	–	–	0.09**	0.19 (0.07)	0.08**	0.19 (0.07)
Acquaintance (between)	–	–	− 0.01	− 0.03 (0.07)	–	–
Gender (between)	–	–	− 0.02	− 0.03 (0.08)	–	–
Student (between)	–	–	− 0.25***	− 0.59 (0.10)	− 0.20***	− 0.47 (0.07)
Age (between)	–	–	− 0.07	− 0.01 (0.00)	–	–
Target (within)	–	–	− 0.34***	− 0.69 (0.02)	− 0.34***	− 0.69 (0.02)
Behavior (within)	–	–	− 0.44***	− 0.31 (0.01)	− 0.44***	− 0.31 (0.01)
Linear effect regressed onto						
Alcohol (between)	–	–	− 0.10**	− 0.05 (0.02)	− 0.10**	− 0.05 (0.02)
Acquaintance (between)	–	–	− 0.00	− 0.00 (0.02)	–	–
Gender (between)	–	–	− 0.05	− 0.03 (0.02)	–	–
Student (between)	–	–	0.17***	0.10 (0.03)	0.11**	0.07 (0.02)
Age (between)	–	–	0.06	0.00 (0.00)	–	–
Target (within)	–	–	0.15***	0.09 (0.01)	0.15***	0.09 (0.01)
Behavior (within)	–	–	− 0.36***	− 0.08 (0.00)	− 0.36***	− 0.08 (0.00)
Quadratic effect regressed onto						
Alcohol (between)	–	–	0.08*	0.00 (0.00)	0.08*	0.00 (0.00)
Acquaintance (between)	–	–	0.02	0.00 (0.00)	–	–
Gender (between)	–	–	0.07	0.00 (0.00)	–	–
Student (between)	–	–	− 0.11*	− 0.01 (0.00)	− 0.05	− 0.00 (0.00)
Age (between)	–	–	− 0.04	− 0.00 (0.00)	–	–
Target (within)	–	–	− 0.14***	− 0.00 (0.00)	− 0.07***	− 0.00 (.00)
Behavior (within)	–	–	0.25***	0.01 (0.00)	0.35***	0.01 (0.00)

	Fit index	Δ	Fit index	Δ	Fit index	Δ
CFI	0.90	–	0.91	.01	0.92	0.01
RMSEA	0.16	–	0.09	− 0.07	0.10	0.01
SRMR (between)	–	–	0.04	–	0.04	0.00
SRMR (within)	–	–	0.04	–	0.04	0.00
χ^2^	18,445.50***	–	15,869.13***	− 2576.37	15,846.50***	− 22.63

*p < 0.05. **p < 0.01. ***p < 0.001

### Unconditional Model

The unconditional model tested the overall intercept, linear effect, and quadratic effect for consent perceptions. The overall intercept represents the latent mean of initial consent perceptions reported after the first segment of the vignette across all participants; it also tested whether the grand mean for consent perceptions was significantly different from zero at this time point. The overall linear and quadratic effects tested whether there was linear or quadratic change in consent perceptions over the course of the vignette; a positive coefficient indicates that the change increased over time, and a negative coefficient indicates it decreased.

The unconditional model indicated that the grand mean in consent perceptions at the first vignette segment was significantly greater than zero. The overall positive linear effect in consent perceptions indicated that participants perceived that the fictional targets were significantly more likely to be willing to engage in sexual activity over the course of the vignette, and the overall negative quadratic effect indicated that this rate of change significantly lessened over the course of the vignette. Based on the significant χ^2^ test statistic, the unconditional model did not fit the data well. While the CFI (0.90) and RMSEA (0.16) for this model did not meet recommended cut-offs, the SRMR (0.07) did.

### Conditional Model (Full)

The conditional model tested whether the patterns shown by the overall intercept, linear effect, and quadratic effect varied by between- and within-person differences. Significant effects of predictors on the intercept indicate differences in consent perceptions after the first vignette segment, while significant effects of predictors on the linear or quadratic effects indicate differences in slopes or rates of changes over the course of the vignette.

The full conditional model added four between- and two within-person predictors variables, as well as one covariate. This model fit the data better than the unconditional model. The χ^2^ test statistic was significantly lower than that of the unconditional model, and the fit indices improved.

Consistent with our hypotheses, predictors of consent perceptions and change in these perceptions included student status, the alcohol manipulation, target’s sex, and type of behavior. Further, the effects for student status were significant even when controlling for the effects of age. However, our hypotheses regarding the interpersonal context manipulation and sex of the participant were not supported; neither of these variables significantly predicted differences in latent means or rates of change over time.

### Conditional Model (Reduced)

**Fig. 1:**
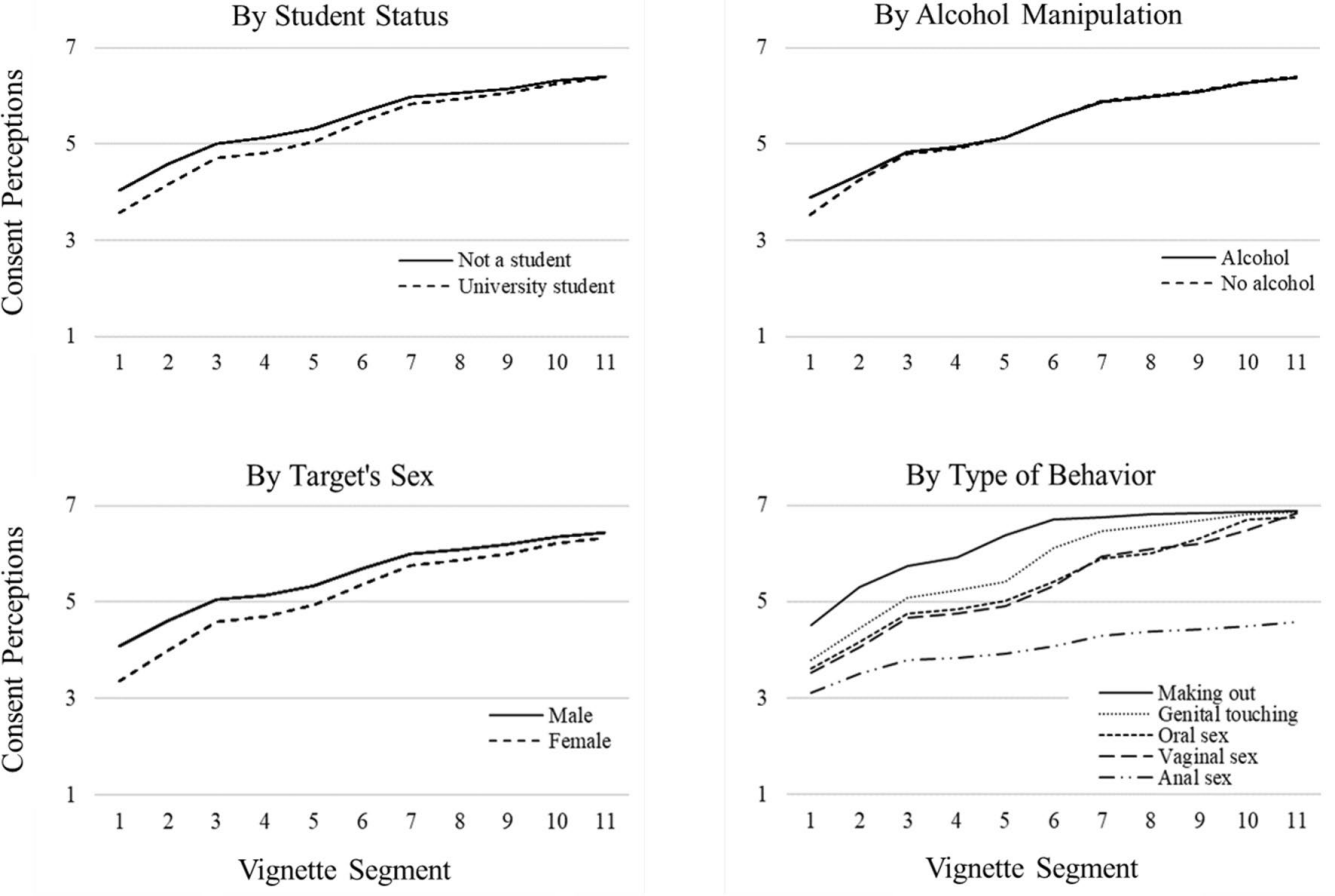
Significant differences in intercepts and in patterns of changes in consent perceptions over the course of the vignette. There were between-person differences by student status and alcohol manipulation as well as within-person differences by target’s sex and type of behavior

Significant predictors of the intercept, linear effect, and quadratic effect from the full model were retained in the reduced conditional model; the other predictor variables were removed from the model. Specifically, the reduced conditional model included two between-person variables (i.e., student status and the alcohol manipulation) and both within-person variables (i.e., target’s sex and type of behavior). The fit indices were similar for the reduced and full conditional models, and the χ^2^ test statistic was not significantly different, Δχ^2^(24) =  − 22.63, *p* = 0.541. Because the reduced model did not worsen data-model fit and it was the more parsimonious model, we interpreted its effects in detail.^1^ Figure 1 depicts the associations between vignette progression and consent perceptions by each of the four predictors included in the reduced conditional model.

#### Between-Person Predictors

Regarding student status, university students (*M* = 3.55, SD = 1.55) perceived the targets as less likely to be willing to engage in sexual activity at the first vignette segment compared with participants who were not currently university students (*M* = 4.02, SD = 1.40), *β* =  − 0.20, *p* < 0.001. Student status also significantly predicted linear rates of change in consent perceptions. Compared with non-students, the linear effect of consent perceptions was steeper for university students, *β* = 0.11, *p* = 0.001, but the quadratic effect was not significant, *β* =  − 0.05, *p* = 0.151. In sum, non-students initially perceived that the targets were more likely to be willing to engage in sexual activity than students; however, the rate of change in consent perceptions was greater for students—such that there were not mean differences by the end of the vignette.

Regarding the alcohol manipulation, participants in the alcohol condition (*M* = 3.90, SD = 1.48) perceived the targets as slightly more likely to be willing to engage in sexual activity at the first vignette segment compared with those in the non-alcohol condition (*M* = 3.52, SD = 1.54), *β* = 0.08, *p* = 0.006. Alcohol condition also significantly predicted linear and quadratic rates of change in consent perceptions. Compared with those in the non-alcohol condition, the linear effect of consent perceptions was less steep for those in the alcohol condition, *β* =  − 0.10, *p* = 0.003, but this effect increased over time, *β* = 0.08, *p* = 0.022. Thus, when participants read that one of the targets accepted a drink from the other target, they perceived greater willingness to engage in sexual activity than when they read that the drink was rejected; however, this difference between conditions quickly dissipated within the first few segments of the vignette.

#### Within-Person Predictors

Regarding target’s sex, participants initially perceived the female target (*M* = 3.34, SD = 1.58) as less likely to be willing to engage in sexual activity than the male target (*M* = 4.06, SD = 1.37) at the first vignette segment, *β* =  − 0.34, *p* < 0.001. Target’s sex also significantly predicted linear and quadratic rates of change in consent perceptions. The linear effect of consent perceptions for the female target was steeper than it was for male targets, *β* = 0.15, *p* < 0.001, and this effect lessened over time, *β* =  − 0.07, *p* < 0.001. As such, participants initially perceived that male targets were more likely to be willing to engage in sexual activity than female targets; however, this difference between conditions decreased over the course of the vignette.

Regarding type of behavior, participants initially perceived the targets as less likely to be willing to engage in incrementally more intimate sexual behaviors at the first vignette segment, *β* =  − 0.44, *p* < 0.001: making out (*M* = 4.50, *SD* = 1.37), genital touching (*M* = 3.79, *SD* = 1.47), oral sex (*M* = 3.61, *SD* = 1.48), vaginal sex (*M* = 3.52, *SD* = 1.48), and anal sex (*M* = 3.11, *SD* = 1.46). Type of sexual behavior also significantly predicted linear and quadratic rates of change in consent perceptions. Compared with relatively less intimate sexual behaviors, the linear effect of consent perceptions was less steep for incrementally more intimate sexual behaviors, *β* =  − 0.36, *p* < 0.001, but this effect increased over time, *β* = 0.35, *p* < 0.001. Therefore, participants initially perceived that targets were more likely willing to engage in relatively less intimate sexual behaviors, and the differences between four types of sexual behavior (i.e., making out, genital touching, oral sex, and vaginal sex) dissipated by the end of the vignette; however, the rate of change for consent perceptions regarding anal sex was far less steep than the other sexual behaviors such that differences between this behavior and the others grew over the course of the vignette.

## Discussion

In 11 segments, our vignette depicted the process of two fictional targets transitioning from flirting in a public setting to having sex in a private setting. We found that participants perceived the targets as more likely to be willing to engage in sexual activity as consent cues—communicative and contextual—accumulated. In the beginning of the vignette, participants on average were “not sure” whether the two targets would be willing to engage in sexual activity; however, they perceived that the targets were significantly more likely to be willing with each new consent cue presented until the behavior happened. This finding is consistent with several qualitative studies that have indicated people perceive consent communication as a process comprising multiple cues (Beres, 2010; Humphreys, 2004; Jozkowski et al., 2018). Our study helps verify this qualitative work and suggests that an accumulation of subtle cues may be perceived by people as indicating consent in the absence of an explicit affirmative communication of consent. We also corroborated previous research regarding four constructs that have been identified as relevant to sexual consent—each of the significant effects was in the directions of our hypotheses. Two other constructs that we predicted would be relevant based on previous research were not significantly associated with consent perceptions in the present study.

First, participants’ sex did not significantly predict differences in consent perceptions or changes in these perceptions over time. Studies have shown that males are more likely than females to perceive targets in vignettes as willing to engage in sexual activity (e.g., Humphreys, 2007; Margolin, 1990). However, a notable distinction between the present study and previous ones is that there were not indicators of refusal or discomfort in the current vignette. By writing a vignette in which both targets were reciprocally engaging in sexual behavior without any indication of refusal or discomfort, participants likely developed their consent perceptions in the absence of any perceived power differentials or uneasiness. And by manipulating the sex of the targets across conditions, there was no evidence in the current vignette of the traditional sexual script that males are the initiators of sex and females the gatekeepers. Together, these egalitarian aspects of the current vignette may have negated sex differences in consent perceptions that have been found in previous studies. Further, recent evidence suggests that gender’s association with sexual consent may be overshadowed when accounting for within-person differences such as type of sexual behavior (Willis et al., 2021a).

Second, we found university students to be more hesitant than non-students to perceive the fictional targets as willing to engage in sexual activity, which might reflect the emphasis that consent education programs have placed on college campuses. Indeed, university students are more likely than non-students to have had formal education of sexual consent, which often teaches that sexual consent should not be assumed without affirmative—even verbal—communication (Curtis & Burnett, 2017; Willis & Jozkowski, 2018). Our study was one of the first to directly compare university students and non-students; most of the previous literature relies on samples that exclusively comprised students (Willis et al., 2019a).

Third, we found that our manipulation of whether one of the targets accepted or rejected an alcoholic drink in the vignette significantly predicted differences in consent perceptions and changes in these perceptions over time. But as evidenced by the relatively small effect sizes, this manipulation was weak; we only included one mention of alcohol, which was at the beginning of the vignette. Nonetheless, the effects of alcohol should be considered in future research on sexual consent. Similar to past reports (Jozkowski et al., 2018), we found that participants perceived targets as more likely to be willing to engage in sexual activity if one had just accepted a drink from the other. It may also be that participants believed the targets were more obligated to have sex if they had accepted a drink, which can similarly influence consent perceptions (Jozkowski et al., 2017). This effect only existed when participants first learned alcohol was present; differences in consent perceptions between the alcohol conditions quickly disappeared. The brief mention of alcohol in this study may not have been enough to produce lasting effects.

Fourth, we did not find any effects of interpersonal context—as manipulated in this study—on the development of participants’ perceptions of sexual consent. Participants did not perceive targets who had just met to be any less willing to engage in sexual activity than those who had been friends for a few weeks. There is evidence that the more important distinction regarding sexual consent is whether people have engaged in sex before (Marcantonio et al., 2018; Monson et al., 2000; Righi et al., 2019). In our vignette, there were no signs that the targets in either condition had a sexual precedent; if there had been, participants may have perceived the process of consent differently. Specifically, perceptions of sexual encounters between acquaintances might vary based on whether or how frequently they had previously had sex with each other.

Fifth, participants’ consent perceptions varied based on the sex of the fictional target they were evaluating. Similar to previous findings (e.g., Humphreys, 2007), people initially perceived the female target to be less willing than the male target to engage in sexual activity. However, participants’ consent perceptions of the female target increased more rapidly than they did for the male target—such that there were no longer differences by the point in the vignette that the targets engaged in a sexual behavior. It may be that the reciprocal advances between the two targets encouraged participants to perceive that first intimate contact to be consensual. That differences in consent perception by target’s sex dissipated around the point of consensual sexual behavior could have important implications for consent communication. For example, people may be reluctant to perceive a female’s consent until she first engages in a seemingly consensual sexual behavior. Because the process of consent should be ongoing, problems might arise if that initial consensual behavior is used to assume consent to other sexual behaviors—especially because males are more likely to perceive consent as a discrete event (Humphreys, 2004; Jozkowski et al., 2018). As our own findings suggested, perceiving consent to one behavior does not equate to perceiving consent to all sexual behaviors.

Finally, the type of sexual behavior in question demonstrated the largest effect sizes and significantly predicted differences in consent perceptions as well as changes in these perceptions over time. Participants generally shifted from being unsure of the targets’ sexual consent to thinking they were definitely willing, but this transition occurred more rapidly for some sexual behaviors. Predictions that making out would be consensual preceded those for genital touching—which preceded those for oral or vaginal sex. Indeed, depictions of these behaviors preceded each other in the vignette itself; however, differing trends between types of behavior were evident before any sexual behaviors occurred between the fictional targets. Therefore, these effects likely are not due solely to the order of events inherent to the vignettes. Findings regarding anal sex starkly contrasted those of the other sexual behaviors; participants were never comfortable assuming the targets’ consent for anal sex. Because anal sex—at least for heterosexual couples—is relatively less common (McBride & Fortenberry, 2010) and people are more likely to report that they would never consent to anal sex compared with other sexual behaviors (Jozkowski, Peterson, et al., 2014), it may not be socially acceptable to perceive somebody as willing to engage in anal sex without explicit verbal cues.

### Implications

In sum, many of the constructs implicated in previous research on sexual consent were relevant for the development of consent perceptions in the present study. Further, our data indicated that the same consent cues can be interpreted differently based on individual differences and contexts. That people take these details into consideration when determining whether certain cues reflect another person’s willingness to engage in sexual activity suggests the process of sexual consent is not as straightforward and simple as “yes means yes.” Contrasting affirmative conceptualizations of sexual consent, our study corroborated previous research that has indicated people perceive and interpret subtle communication and situational factors when determining whether potential partners might agree to sexual behavior (Beres, 2010; Jozkowski et al., 2018).

We urge researchers to investigate and educators to emphasize the nuances of sexual consent—especially that perceptions of sexual consent vary across people as well as within people across contexts. For example, we found that the same cues were not unilaterally perceived as being indicators of willingness to engage in sexual activity for all people or all circumstances. Rather, individual differences (e.g., student status; sex of target) and situational contexts (e.g., alcohol use; type of sexual behavior) differentiated sexual consent perceptions. Such nuances are key to understanding sexual consent and should continue to be examined empirically.

### Limitations and Future Directions

Even though all significant differences in latent means were in the hypothesized directions, the associations with linear and quadratic effects were consistently different than our theory-based predictions. It seemed that ceiling effects hampered our ability to test our expectations. Specifically, most participants across conditions perceived the targets as definitely likely to be willing to engage in sexual activity (except for anal sex) by the 11th segment. Because there were latent mean differences in consent perceptions at the first segment and all conditions ended up in the same place, the slopes for those conditions with lower perceptions at the intercept were necessarily higher than the slopes of their respective conditions. That we designed the vignette segments to exclusively provide consent cues (and not also indicators of ambivalence or refusal) may have contributed to this ceiling effect by increasing demand characteristics.

Regarding the generalizability of our findings, this study was only able to assess consent perceptions for a very specific sexual encounter—one that exclusively included nonverbal consent communication cues, mixed-sex targets, a prototypical progression from making out to intercourse, a single mention of alcohol, and targets without sexual precedent. It may be that the development of sexual consent perceptions would be different if we had included verbal consent cues or refusal cues, same-sex targets, a different order of sexual behaviors, apparent effects of intoxication cues, or targets that had engaged in sex before. All of these variations should be pursued in future research on people’s sexual consent perceptions.

Finally, to provide evidence for the ecological validity of the present study’s findings based on fictional vignettes, future studies could employ other methodologies to investigate sexual consent perceptions. For example, experimentally manipulating participants’ sexual arousal is a way to partially mediate concerns regarding the discrepancy between responding to a hypothetical scenario and actually experiencing the process of sexual consent. Indeed, previous research has found that sexual arousal can influence sexual consent perceptions (Benbouriche et al., 2019). Further, experience sampling methodology could be used to assess in-the-moment consent perceptions as has been done with self-reports of internal consent feelings and external consent communication (Willis et al., 2021b). By collecting daily data on people’s sexual consent perceptions over time, researchers would be able to investigate the trends in people’s lived sexual consent process and examine the contexts in which people’s perceptions of another person’s willingness might be associated with their own experiences or communication of willingness.

## Conclusion

The main objective of the present study was to examine changes in consent perceptions across 11 staggered segments of a vignette that depicted a consensual sexual encounter between two targets. The latent growth curve models in the present study examined overall patterns of change and differences in consent perceptions by between- and within-person variables. We found that initial consent perceptions and trends over the course of the vignette varied by whether the participant was a university student, whether an alcohol drink was accepted or rejected in the vignette, the sex of the fictional target, and the type of sexual behavior. Because previous research has relied on retrospective accounts of consent perceptions, this study provided an important glimpse at the process of developing sexual consent perceptions that had been lacking in the extant literature. Researchers should continue to examine the nuanced process of accumulating subtle cues people may perceive as sexual consent.

## Data Availability

The data underlying this article will be shared on reasonable request to the corresponding author.

## Vignette Version: Kim first; Just met; No alcohol

1. Kim and Mike are at a bar with friends when they meet for the first time. They begin talking and are enjoying each other's company. Kim offers Mike a drink. Mike says he’s not drinking tonight.
2. Both are flirting with each other. Mike touches Kim's arm while he laughs at something she says.
3. Kim invites Mike to get a ride home with her. Mike accepts.
4. Once at Kim's place, they decide to watch a movie together. Kim starts the movie and then sits down next to Mike so that their legs are touching.
5. A few minutes into the movie, Mike reaches for Kim's hand. Kim smiles and places her hand in his.
6. A while later, Kim leans in to kiss Mike. They start making out. Kim reaches her hand under Mike's shirt. Mike pulls her closer.
7. They take off their own shirts, and Kim undoes her bra. Kim then leads Mike to her bedroom. Mike follows.
8. They both take off their own pants before getting into Kim's bed. Kim and Mike start making out again, Kim on top.
9. Kim kisses Mike's neck and continues to do so from his chest to his stomach. Mike lifts his butt for Kim to take off his underwear.
10. Kim takes off Mike's underwear and starts to give him a blowjob. After a while, Kim crawls up to her nightstand and grabs a condom.
11. Mike takes the condom, opens it, and puts it on himself. Kim and Mike begin to have sex.

## Vignette Version: Mike first; Friends; Alcohol

1. Mike and Kim have been friends for a few weeks. Tonight, they are hanging out at a bar and are enjoying each other's company. Mike offers Kim a drink. Kim accepts.
2. Both are flirting with each other. Kim touches Mike's arm while she laughs at something he says.
3. Mike invites Kim to get a ride home with him. Kim accepts.
4. Once at Mike's place, they decide to watch a movie together. Mike starts the movie and then sits down next to Kim so that their legs are touching.
5. A few minutes into the movie, Kim reaches for Mike's hand. Mike smiles and places his hand in hers.
6. A while later, Mike leans in to kiss Kim. They start making out. Mike reaches his hand under Kim's shirt. Kim pulls him closer.
7. They take off their own shirts, and Mike undoes Kim's bra. Mike then leads Kim to his bedroom. Kim follows.
8. They both take off their own pants before getting into Mike's bed. Mike and Kim start making out again, Mike on top.
9. Mike kisses Kim's neck and continues to do so from her breasts to her stomach. Kim lifts her butt for Mike to take off her underwear.
10. Mike takes off Kim's underwear and starts to go down on her. After a while, Mike crawls up to his nightstand and grabs a condom.
11. Kim takes the condom, opens it, and puts it on Mike. Mike and Kim begin to have sex.

## Footnotes

1 The effects reported did not change in significance, magnitude, or direction when conducting separate analyses with a subsample that only included participants aged 18–30.
